# Does Intraoral Scanning at the Subgingival Finish Line Affect the Accuracy of Interim Crowns?

**DOI:** 10.3390/jfb16090309

**Published:** 2025-08-27

**Authors:** Young-Tak Son, Keunbada Son, Ji-Min Lee, Kyu-Bok Lee

**Affiliations:** 1 Department of Dental Science, Graduate School, Kyungpook National University, Daegu 41566, Republic of Korea; dudxkr741@naver.com (Y.-T.S.); wlals9408@naver.com (J.-M.L.); 2 Advanced Dental Device Development Institute, Kyungpook National University, Daegu 41566, Republic of Korea; oceanson@knu.ac.kr; 3 Department of Prosthodontics, School of Dentistry, Kyungpook National University, Daegu 41566, Republic of Korea

**Keywords:** subgingival finish line, interim crown, internal surface trueness, marginal and internal fit, correlation, gingival retraction cord

## Abstract

This in vitro study evaluated the internal surface trueness and the marginal and internal fit of interim crowns fabricated from intraoral scanner (IOS; i500, MEDIT, Seoul, Republic of Korea) data, considering variations in subgingival finish line depth and the use of gingival retraction. A right maxillary first molar was prepared using a milled ceramic abutment, with subgingival finish line depths set at 0, 0.25, 0.50, 0.75, and 1.00 mm from the gingival crest. All specimens were scanned with an IOS, both with and without gingival retraction. Interim crowns were designed from the scan data and produced via three-dimensional (3D) printing. Internal surface trueness was measured using 3D inspection software (Geomagic Control X version 2022.0.0; 3D Systems, Rock Hill, SC, USA), while marginal and internal fit were assessed with the silicone replica technique. Data were analyzed using the Mann–Whitney U test and Kruskal–Wallis H test (α = 0.05). In the absence of gingival retraction, internal surface trueness and crown fit decreased significantly with increasing finish line depth (*p* < 0.05). At a 1.00 mm depth without retraction, internal trueness reached 100.1 ± 44.5 µm and marginal fit was 189.1 ± 42.2 µm, both exceeding clinical thresholds. With gingival retraction, trueness and fit remained stable across all depths (*p* > 0.05). At 1.0 mm depth, trueness was 82.0 ± 61.8 µm and marginal fit was 95.2 ± 22.9 µm, both within clinically acceptable limits. A significant correlation was observed between marginal trueness and overall fit when retraction was not performed (*p* < 0.05). These results demonstrate that increasing subgingival finish line depth can significantly reduce intraoral scanning accuracy, resulting in suboptimal interim crown adaptation when gingival retraction is omitted. To achieve clinically acceptable internal trueness and marginal fit, gingival displacement with a retraction cord is recommended during intraoral scanning for subgingival prosthesis fabrication.

## 1. Introduction

Advances in digital dentistry, particularly computer-aided design and manufacturing (CAD/CAM), have made scanners indispensable tools in prosthodontic workflows [1,2]. Despite these advancements, desktop scanners remain susceptible to errors arising from the fabrication and scanning of working casts [3,4,5]. Intraoral scanners (IOSs), by contrast, can minimize such errors during the clinical workflow [6]. Evaluations of IOS accuracy have primarily focused on trueness and precision under a range of clinical scenarios [3,7,8,9,10,11,12,13,14]. However, few studies have assessed the accuracy of prostheses fabricated from virtual working casts featuring subgingival finish lines.

Previous research has investigated the marginal and internal fit of dental prostheses produced using casting, milling, and additive manufacturing techniques [15,16,17,18]. The emergence of additive manufacturing—especially for resin-based materials—has prompted recent studies to examine the adaptation accuracy of 3D-printed dental restorations. Provisional restorations fabricated via in-office workflows have attracted substantial interest due to their roles in preserving soft tissue stability, increasing patient comfort, and potentially affecting the fit of subsequent definitive prostheses. Marginal gaps exceeding 120 µm may cause lesions and lead to prosthesis failure [19,20,21,22]. In addition, fabrication reproducibility has often been assessed by analyzing internal surface trueness [16,17,23,24,25]. Internal surface trueness values below 100 µm are generally considered clinically acceptable, given the required cement space for dental prostheses [26,27].

During fixed prosthesis fabrication, subgingival finish lines of varying depths may be necessary to meet individual patient needs [28,29,30,31]. Biologically, it is critical that the gingival margin does not extend more than 1 mm into the gingival sulcus [32,33]. Subgingival margins exceeding this depth may induce gingival lesions [32,33]. The precision of intraoral scanning at the subgingival finish line is another important consideration. Prior studies have validated the relationship between IOS accuracy and subgingival finish line depth [31,34], demonstrating that scan accuracy decreases as the finish line depth increases. Subgingival scanning is inherently challenged by limited optical access, reduced light reflection from moist or hemorrhagic sulcular tissues, and shadowing from adjacent soft tissue [30,31,32,33,34]. These barriers impede data acquisition when the finish line is located deep within the sulcus. Scan trueness reportedly declines significantly when the finish line is positioned more than 0.5 to 1.0 mm subgingivally [34]. To address these limitations, gingival displacement techniques—such as retraction cords or astringent-impregnated materials—are employed to temporarily expose the finish line and improve scanning accuracy. Several studies have confirmed that gingival retraction enhances marginal adaptation and reduces error propagation in digital impressions involving subgingival margins [30,31,34,35]. Nonetheless, limited evidence exists regarding the impact of inaccurately scanned data from deeper subgingival depths on the fabrication accuracy of dental prostheses. While interest in the effects of subgingival finish line depth on IOS accuracy has increased, most studies have focused on surface deviation or virtual data analysis alone [30,34]. However, in clinical practice, the validity of scan data must ultimately be confirmed by the adaptation of fabricated restorations. Few investigations have systematically explored whether scan inaccuracies—particularly in the absence of gingival retraction—are reflected in the marginal and internal fit of interim crowns. Addressing this knowledge gap is crucial for understanding the clinical implications of subgingival scanning and for optimizing digital workflows. At present, validating IOS accuracy requires fabricating interim crowns from scanned data and subsequently assessing the fit of these crowns.

Because IOSs are optical devices, they can only capture visible surfaces. For enhanced visibility in clinical scenarios, gingival displacement with a retraction cord is often necessary [34,35]. Previous research has demonstrated that gingival displacement increases IOS accuracy [30]. However, the accuracy of abutment scanning with and without gingival retraction during prosthetic fabrication remains unexamined.

Few studies have examined whether inaccuracies in intraoral scan data are transmitted to the adaptation of fabricated restorations. In this study, interim crowns were fabricated and quantitatively analyzed to assess internal surface trueness and marginal and internal fit. This approach enabled evaluation of how scan data quality—under different subgingival finish line depths and gingival retraction conditions—affects crown adaptation. Such an integrated methodology underscores the need to optimize digital workflows in fixed prosthodontics.

Accordingly, this study aimed to evaluate the effects of subgingival finish line depth and the presence or absence of gingival retraction on the marginal and internal fit and the internal surface trueness of interim crowns.

## 2. Materials and Methods

**Figure 1:**
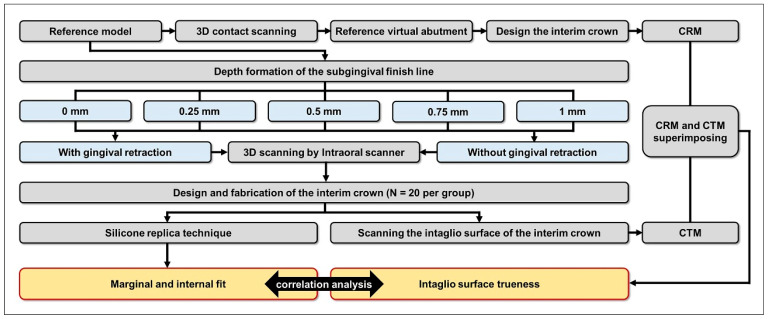
Assessment of interim crown marginal and internal fit and internal surface trueness, according to subgingival finish line depth, with and without gingival retraction.

To fabricate the working casts, the subgingival finish line was established based on the vertical distance from the gingival crest to the finish line [30]. To test virtual model production, the fabricated models were scanned both with and without gingival displacement. Interim crowns were then designed based on these test virtual models and subsequently 3D-printed to enable assessment of marginal and internal fit. Additionally, a CAD test model (CTM) was created by scanning the internal surface of each 3D-printed interim crown. The interim crown designed using the reference virtual abutment—obtained by tactile scanning of the abutment—was designated as the CAD reference model (CRM). Internal fabrication reproducibility was determined by comparing the CRM and CTM (Figure 1).

The maxillary first molar reference model was prepared using a milling machine (EZIS HM; DDS, Seoul, Republic of Korea). Abutments were fabricated from lithium disilicate ceramic (IPS e.max CAD A2 Shade; Ivoclar Vivadent AG, Schaan, Liechtenstein). Preparations included a 1.5 mm reduction in the occlusal direction, a 1.2 mm axial reduction, and a chamfer-shaped finish line. A diamond rotary bur (ZR8850; Komet Dental Gebr. Brasseler GmbH & Co. KG, Lemgo, Germany) was used to polish the abutment surfaces and reduce gloss. Working casts were produced with a 3D printer (Meg-Printer 2; Megagen, Daegu, Republic of Korea). Porcine neck soft tissue was applied to the working cast to simulate the gingiva [34].

Ethical approval from the Institutional Review Board was not required for this in vitro study. The simulated gingival tissues were obtained from the cervical region of porcine specimens (pork neck tissue) purchased from a local butcher in Daegu, Republic of Korea. These tissues originated from pigs slaughtered for human consumption and were not collected specifically for research. As no live animals were used and no biological materials were acquired outside of routine food production, the study was exempt from ethical review. The Institutional Animal Care and Use Committee of Kyungpook National University formally waived the requirement for ethical approval. To determine the required sample size per group, five pilot studies were conducted. Based on results from power analysis software (G*Power v3.1.9.2; Heinrich-Heine-Universität, Düsseldorf, Germany), 20 samples per group were selected (actual power = 80.7%, power = 80%, α = 0.05).

**Figure 2:**
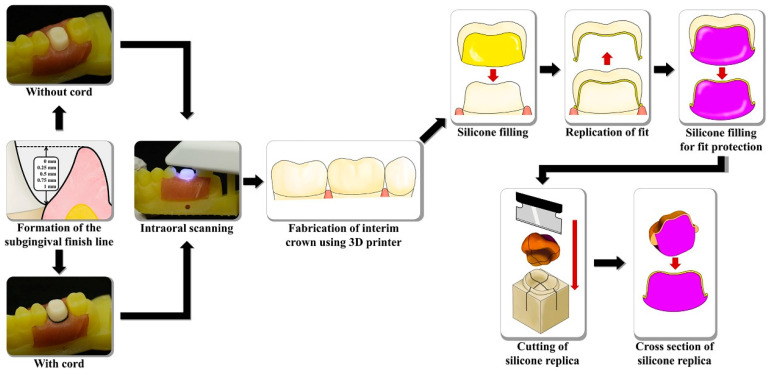
Silicone replica technique used to evaluate marginal and internal fit.

Five standard tessellation language (STL) files for the fabricated abutments were acquired using a tactile scanner (DS10, Renishaw plc, Wotton-under-Edge, Gloucestershire, UK). After alignment and optimization with 3D reverse engineering software (Geomagic Design X version 2022.0.0; 3D Systems, Rock Hill, SC, USA), the five virtual abutment models were merged to create a high-resolution reference virtual abutment. To assess fabrication reproducibility, an interim crown was designed using the reference virtual abutment as the CRM. Following the contact scan of the abutment, it was attached to the 3D-printed working cast, and the vertical distance from the gingival crest to the finish line was established by manipulating the porcine tissues (Figure 2). The subgingival finish line depth was verified using a vernier gauge (500–151-30; Mitutoyo, Takatsu-ku, Japan) and a periodontal probe (William’s probe; Premium Instruments, Ronkonkoma, NY, USA). The measured subgingival finish line depths were as follows: 0.2521 ± 0.0039 mm (0.25 mm group), 0.5008 ± 0.0005 mm (0.5 mm group), 0.7507 ± 0.0008 mm (0.75 mm group), and 1.0006 ± 0.0005 mm (1 mm group). A single IOS (i500; MEDIT, Seoul, Republic of Korea) was used to obtain the test virtual models. No scanning powder was applied. All scans were performed by an experienced operator (Y.-T.S.) under standardized indoor lighting, using the scanner’s standard mode at maximum depth. Twenty scans were completed without gingival displacement, and 20 with gingival displacement. Gingival displacement was performed using a retraction cord (Ultrapak, Ultradent Products Inc., South Jordan, UT, USA), which was placed in the gingival sulcus with a cord packer (Fischers Ultrapak Packer, Ultradent Products Inc., South Jordan, UT, USA). The artificial gingiva was moistened with distilled water to prevent drying, and excess moisture from other regions was removed to optimize scanning conditions. All scanning was performed by a single experienced investigator (Y.-T.S.). Each virtual test model was saved as an STL file.

Interim crowns for all test virtual models were fabricated using dental CAD software (Dental System 2022; 3Shape, Copenhagen, Denmark). During the design process, the following parameters were set: a cement space of 60 µm and a distance of 1 mm from the finish line. Using each test virtual model as a template, 200 interim crowns were designed. For fabrication, a dedicated photocurable resin (RAYDENT C&B; Ray, Seoul, Republic of Korea) was employed with a 3D printer. After printing, each interim crown was cleaned and post-cured according to the manufacturer’s instructions.

**Figure 3:**
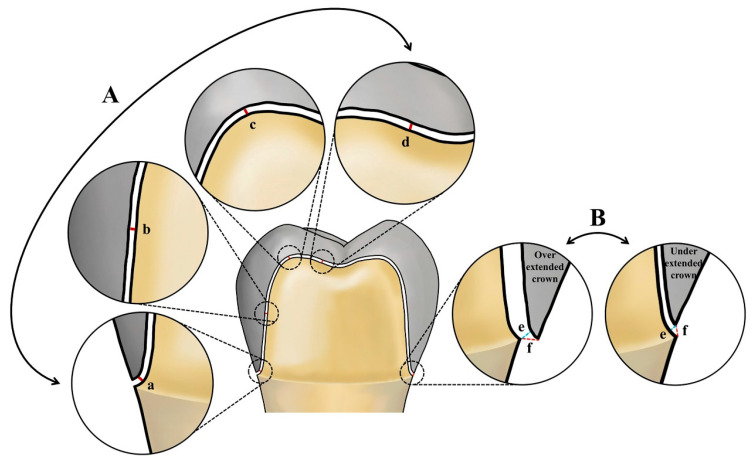
Schematic illustration of measurement points for marginal and internal fit. (A) Internal fit: (a) chamfer gap, (b) axial gap, (c) angle gap, (d) occlusal gap. (B) Marginal fit: (e) marginal gap, (f) absolute marginal discrepancy.

Figure 2 depicts the workflow for evaluating marginal and internal fit. The silicone replica technique was adopted for this assessment. Each interim crown was filled with a highly flowable light body impression material (Aquasil Ultra XLV; Dentsply Detrey GmbH, Konstanz, Germany), seated onto the abutment, and held in place. Constant pressure was applied to the occlusal surface until polymerization was complete. Once the light body material is set, the temporary prosthesis was removed. A medium body impression material of a contrasting color (Aquasil Ultra Monophase; Dentsply Detrey GmbH, Konstanz, Germany) was then layered onto the silicone replica, capturing both internal and marginal fit. After polymerization, the silicone complex was separated from the interim crown. A custom jig, designed for precise sectioning of the silicone replicas, was created based on CRM data using CAD software (SolidWorks 2015; Dassault Systèmes SolidWorks Corp., Waltham, MA, USA) and produced with a 3D printer. The silicone replicas were sectioned along the buccolingual and mesiodistal planes through the center, and the internal and marginal fits were measured using a video microscope system (IMS 1080P; SOMETECH, Seoul, Republic of Korea). Figure 3 presents the measurement results for internal and marginal fit.

The CRM was an interim crown designed using the reference virtual abutment. All interim crowns were created based on the test virtual model, and the internal surfaces were further scanned using an IOS (i500; MEDIT). STL files generated from scanning the internal surfaces of all interim crowns were designated as CTM and used to evaluate fabrication reproducibility [31]. A 3D inspection software (Geomagic Control X version 2022.0.0.; 3D Systems, Rock Hill, SC, USA) was utilized to align and analyze the internal surface data of both CRM and CTM. For comprehensive analysis of internal surface trueness, the evaluation regions were classified as whole, marginal, axial, and occlusal. Initial alignment was performed using the global best-fit function within the 3D inspection software. To enhance alignment accuracy, the internal intaglio surface of the crown was specified as the region of interest (ROI), and final alignment was conducted using point cloud data confined to this area. The sampling ratio was established at 100%, and RMS values were calculated from the shortest distances between corresponding points in the selected regions of CRM and CTM. The calculation formula was as follows (1):

(1) $$RMS=\frac{1}{\sqrt{n}}\cdot \sqrt{\sum_{i=1}^{n}{\left(X_{1,i}-X_{2,i}\right)}^{2}}$$

*X*_1,*i*_ represents the k-th measurement point in CRM, while *X*_2,*i*_ indicates the *i*-th measurement point in CTM. The variable *n* denotes the total number of measured points. The root mean square (RMS) value, calculated as the absolute average distance among all cloud points, quantifies the level of agreement between CRM and CTM.

Statistical analyses were conducted using SPSS Statistics for Windows, version 25.0 (IBM Corp., Armonk, NY, USA). Internal surface trueness, marginal fit, and internal fit data were evaluated at a significance level of α = 0.05. The Shapiro–Wilk test assessed the normality of the data distributions. The Mann–Whitney U test and Kruskal–Wallis H test were applied to compare marginal and internal fit, as well as internal surface trueness, between groups. Pearson’s correlation analysis evaluated the relationship between marginal region trueness and both marginal and internal fit. Where the Kruskal–Wallis test identified significant differences among the five finish line depths (0, 0.25, 0.50, 0.75, and 1.00 mm), post hoc pairwise comparisons were performed using the Bonferroni-adjusted Dunn procedure. The strength of correlations was interpreted based on the absolute value of Pearson’s r coefficient: weak (0.10–0.39), moderate (0.40–0.69), and strong (0.70–0.89). Effect sizes (r for Mann–Whitney U and η^2^ for Kruskal–Wallis H) were calculated, where appropriate, to assess the magnitude of group differences. Results are presented as mean ± standard deviation (SD), with 95% confidence intervals (CI) provided where applicable.

## 3. Results

**Figure 4:**
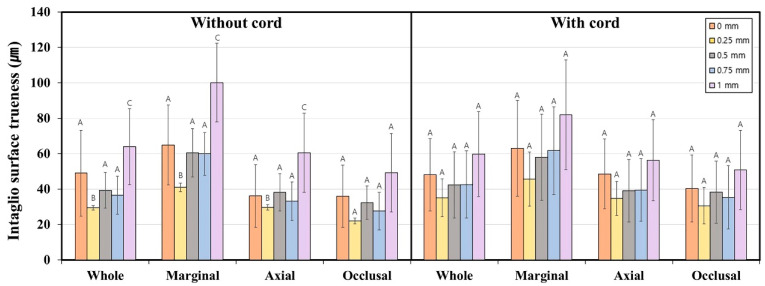
Marginal and internal fit of interim crowns with various subgingival finish line depths, with and without gingival retraction. Identical letters indicate no significant difference between groups (*p* ≥ 0.05).

Figure 4 and Table 1 present a comparison of internal surface trueness for interim crowns featuring subgingival finish lines of varying depths, both with and without gingival displacement. The use of a retraction cord did not significantly influence internal surface trueness (*p* > 0.05). However, in the absence of a gingival retraction cord, finish line depth significantly affected trueness in the whole, marginal, and axial regions (*p* < 0.001). Without retraction, internal surface trueness declined as finish line depth increased, with the poorest result at 1.0 mm. At this depth, marginal region trueness reached 100.1 ± 44.5 μm, surpassing the clinically acceptable threshold. In contrast, when a retraction cord was employed, no significant association was found between finish line depth and internal surface trueness (*p* > 0.05). With gingival retraction, trueness in the marginal region remained below 100 μm for all groups, with the maximum value of 82.0 ± 61.8 μm observed at the 1.0 mm depth.

**Figure 5:**
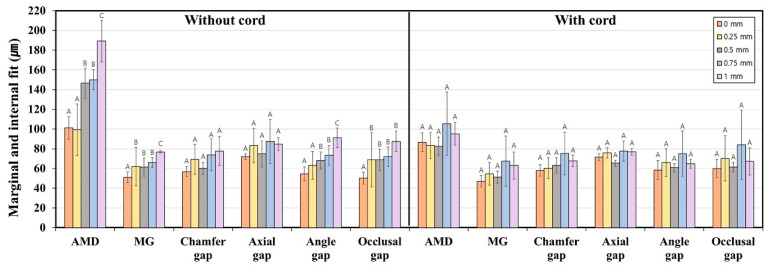
Internal surface trueness of interim crowns with various subgingival finish line depths, with and without gingival retraction. Identical letters indicate no significant difference between groups (*p* ≥ 0.05).

Figure 5 and Table 2 present the marginal and internal fit of interim crowns fabricated with various subgingival finish line depths, both with and without gingival displacement. Significant differences in marginal fit were identified according to the use of a gingival retraction cord in the 0-, 0.75-, and 1 mm depth groups (absolute marginal discrepancy (AMD)), as well as in the 0.5 mm group for AMD and marginal gap (MG) (*p* < 0.05). In particular, the 1.0 mm group exhibited significant differences in both internal fit (axial, angle, and occlusal gap) and marginal fit (AMD) depending on retraction (*p* < 0.05). For the 1.0 mm group without retraction, the AMD and MG measured 189.1 ± 42.2 μm and 76.8 ± 20.2 μm, respectively. When gingival retraction was used, these values decreased to 95.2 ± 22.9 μm and 63.1 ± 27.4 μm. In the absence of gingival retraction, both marginal and internal fit values increased as the finish line depth increased (*p* < 0.05). However, when gingival retraction was applied, marginal and internal fit values did not vary significantly among crowns with different finish line depths (*p* > 0.05).

Table 3 presents the relationship between the trueness of the internal surface at the marginal region and both marginal and internal fit. Without the use of a gingival retraction cord, there was a significant positive correlation between marginal fit (AMD), internal fit (angle and occlusal gap), and marginal region trueness (*p* < 0.05). Conversely, with the application of a gingival retraction cord, no significant correlation was found between marginal fit and marginal region trueness (*p* > 0.05; Table 3). Nonetheless, a weak correlation was detected between internal fit and marginal region trueness in this condition (*p* < 0.05).

## 4. Discussion

This study found that increasing the subgingival depth of the finish line adversely affected both the marginal and internal fit, as well as the internal surface trueness, of interim crowns. The results indicate that deeper margins may reduce scan accuracy and compromise crown adaptation. In contrast, gingival displacement exerted minimal influence on internal surface trueness, implying that this factor alone does not substantially impact the internal surface characteristics relevant to crown adaptation under the tested conditions.

Previous research has proposed that internal surface trueness should be less than 100 µm for clinical acceptability [26,27]. Son et al. [30] reported the poorest internal surface trueness in the marginal region—50.8 ± 11.9 µm—when gingival retraction was omitted and the subgingival finish line depth was 0.5 mm. In our study, the marginal region exhibited a trueness of 60.5 ± 27.4 µm without gingival retraction at a 0.5 mm subgingival finish line depth, while the greatest deviation (100.1 ± 44.5 µm) occurred at a 1 mm depth. However, direct comparisons with previous studies are challenging due to variations in study designs. Our findings suggest that internal surface trueness declines when prostheses with a subgingival finish line are fabricated using IOSs, which aligns with previous reports.

In the absence of retraction cord, this study observed a clear trend: as the subgingival finish line depth increased, internal surface trueness worsened. This decline in scanning accuracy primarily results from limited optical access, inherent limitations of current intraoral scanner (IOS) technology, and interference from surrounding soft tissues [4,8,11,13,14,31,34,36]. In deeper sulcular regions, scanner light cannot adequately reach or reflect from the finish line due to fluid accumulation (such as saliva, blood, or crevicular fluid) and reduced visibility, collectively hindering image acquisition and signal return. Additionally, the optical principles utilized in most IOS systems—such as structured light and confocal technologies—perform less effectively in geometrically complex or shadowed areas, particularly when the finish line extends beyond 0.5 to 1.0 mm subgingivally [31,34]. Without gingival retraction, gingival tissue may collapse over or cover the margin, further impeding accurate data capture and increasing the risk of surface registration errors. These combined factors contribute to discrepancies between the scanned model and the actual tooth preparation, ultimately diminishing the marginal and internal fit of the final restoration. Nedelcu et al. [36] compared IOS accuracy for supragingival and subgingival finish lines, showing that subgingival finish lines generally yielded lower measurement accuracy. Furthermore, lower scan accuracy of the working cast may result in a final prosthesis contour that is unnecessarily large or small. A previous study reported that IOS trueness exceeds 100 µm when the subgingival finish line depth is 0.5 mm or greater [34]. Consistent with these findings, our study demonstrated that greater subgingival finish line depths correlated with increased internal surface trueness deviations for interim crowns fabricated without gingival retraction. This outcome suggests that prosthetic design may be suboptimal, as IOS accuracy decreases with greater subgingival depth. Margins that are too short or too long, or marginal fits that fall outside the clinical range, can promote periodontal lesions. In our study, when marginal region trueness exceeded 100 µm at a 1.0 mm subgingival depth without gingival retraction, the absolute marginal discrepancy increased to 189.1 ± 42.2 µm. Marginal discrepancies above 120 µm are typically considered beyond the clinically acceptable threshold for crown margins [19]. Such deviations may increase cement film thickness, promote marginal leakage and plaque accumulation, and elevate the risk of secondary caries and periodontal inflammation [20,21]. Additionally, larger internal fit discrepancies, especially in the angle and occlusal regions, can hinder prosthesis seating, necessitate more chairside occlusal adjustment, or lead to premature failure [22]. These results emphasize the importance of achieving high scan trueness to optimize prosthesis adaptation in clinical practice, particularly for subgingival margins. Therefore, careful attention is required during both scanning and fabrication.

A previous study observed significant variation in internal surface trueness at the subgingival finish line, depending on whether gingival retraction was used [30]. That study found a trueness of 50.8 ± 11.9 µm without gingival retraction and 41.3 ± 5.8 µm with gingival retraction [30]. In contrast, our results indicate that gingival displacement did not appreciably affect the internal surface trueness of the prosthesis. However, when a retraction cord was not applied, trueness at a 1 mm depth exceeded 100 µm, surpassing the clinically acceptable range, whereas with gingival displacement, trueness was 82 µm and within acceptable limits. Further research is needed to clarify the effect of gingival displacement on internal surface trueness in dental prostheses.

Multiple techniques have been explored to accurately scan subgingival finish lines for dental prosthesis fabrication [37,38,39]. Prostheses should be fabricated after intraoral scanning using established methods, and the suitability of each method should be evaluated by assessing marginal and internal fit. McLean et al. [19] recommended that prosthesis fit should remain below 120 µm for clinical acceptability. Markarian et al. [40] reported that the marginal fit of prostheses with a subgingival finish line was 149.78 µm, which exceeds the clinically acceptable range. However, when gingival interference was absent during scanning, a marginal fit of 94 µm was achieved. In our study, subgingival finish lines of 0.75 mm depth or greater resulted in a marginal fit of 146.6 µm in AMD. With gingival retraction, the marginal fit improved to 82.6 µm, which is within the clinical threshold. These findings suggest that omitting a gingival retraction cord may compromise the fit of prostheses with subgingival finish lines fabricated via intraoral scanning. The present study also confirmed that both marginal fit (as measured by AMD and MG) and internal fit (assessed at the angle and occlusal gap) deteriorated as subgingival finish line depth increased. In contrast, when gingival retraction was performed, neither marginal nor internal fit exceeded the clinical threshold, regardless of measurement depth or location. Thus, gingival retraction is recommended for prosthetic cases involving subgingival finish lines.

Additionally, our study revealed that, without gingival retraction, the internal surface trueness reached its highest value in the marginal region, with significant differences observed as margin depth varied. Accordingly, we evaluated the correlation between marginal and internal fit and trueness in the marginal region. Jang et al. [41] measured fit and trueness in dental prostheses, reporting a positive correlation between internal trueness and internal fit when prostheses were fabricated using in-lab (open type) methods. In our study, when gingival retraction was not performed, a positive correlation was observed between marginal fit (AMD), internal fit (angle and occlusal gap), and marginal region trueness. These results indicate that inaccuracies in the marginal region during interim crown fabrication can adversely affect overall fit. Thus, careful attention is needed to achieve appropriate internal trueness and both marginal and internal fit when fabricating prostheses with subgingival finish lines.

Prior studies have identified that IOS errors can arise from software updates [42], calibration [43], and operator variability [44]. In this study, a single experienced investigator performed all scans, ensured software was up to date, and maintained continuous calibration to minimize these variables. Interim crowns were fabricated using the digital light processing (DLP) technique, which previous research has shown to match or exceed the accuracy of stereolithography [45,46]. Various studies have sought to replicate clinical conditions experimentally. For example, Son et al. [30] simulated gingiva by adding red pigment to transparent silicone, while Michou et al. [47] reproduced gingival anatomy by mounting extracted teeth on a visible light-cured resin base mimicking tooth-supporting tissue optical properties. Marotti et al. [38] and Choi et al. [48] used pig tissue obtained from animals slaughtered for human consumption to simulate the clinical environment. In this study, a subgingival finish line was created using tissue from the neck of a slaughtered pig to replicate clinical conditions. Nevertheless, the artificial gingiva formed did not perfectly reproduce the oral cavity or fully account for anatomical factors such as lips, saliva, and cheeks.

This study had several limitations that should be acknowledged when interpreting the results. First, the investigation was performed entirely in vitro, which does not replicate the complex and variable environment of the oral cavity, such as patient movement, salivary presence, and soft tissue dynamics. Although porcine soft tissue was employed to mimic the subgingival setting, this model does not fully capture the anatomical, optical, or biomechanical attributes of human gingiva. Additionally, only one intraoral scanner, 3D printer, and resin material were used, potentially limiting the extrapolation of these results to other digital systems or workflows. Collectively, these constraints reduce the clinical applicability of the findings. To address these limitations, future research should utilize a broader range of intraoral scanners and fabrication devices and include in vivo validation under diverse clinical scenarios, including varying sulcular depths, gingival biotypes, and margin positions. Moreover, it is recommended to develop advanced soft tissue simulation models that more accurately reproduce the histological and optical properties of human gingiva, thereby enhancing the translational relevance of in vitro studies.

## 5. Conclusions

Within the constraints of this in vitro study, several key conclusions emerge. Increasing the subgingival finish line depth without gingival retraction led to a deterioration in both the marginal and internal fit of interim crowns. Specifically, marginal discrepancies exceeded 120 µm at depths of 0.5 mm and greater, while internal surface trueness in the marginal region surpassed 100 µm at a depth of 1.0 mm. In contrast, gingival retraction kept both measures within clinically acceptable thresholds at all tested depths. These findings indicate that subgingival finish lines deeper than 0.5 mm may compromise intraoral scanning accuracy unless soft tissue management is implemented. Clinically, when subgingival finish lines are necessary within biologic limits, the use of gingival displacement techniques such as retraction cords is recommended to achieve accurate digital impressions. Future in vivo research incorporating various scanner systems and tissue conditions is needed to validate and broaden the applicability of these results.

## Figures and Tables

**Table 1:** Internal surface trueness by subgingival finish line depth with or without gingival displacement cord.

Region Type	Finish Line Depth (mm)	Cord Type	Mean	SD	95% CI		*p* *	Without Cord; *p* **	With Cord; *p* ***
					Lower	Upper			
Whole region	0	Without cord	49.0	48.6	31.2	69.6	0.421	<0.001 ** (1 mm > 0.75, 0.5, 0 mm > 0.25 mm)	0.334
		With cord	48.1	40.9	28.9	67.3			
	0.25	Without cord	29.4	2.6	28.2	30.5	0.243		
		With cord	35.1	21.5	25.0	45.2			
	0.5	Without cord	39.3	20.2	29.9	48.8	0.754		
		With cord	42.3	37.4	24.8	59.9			
	0.75	Without cord	36.5	21.4	26.5	46.5	0.532		
		With cord	42.6	37.8	24.9	60.3			
	1.0	Without cord	64.0	42.9	43.9	84.1	0.771		
		With cord	59.8	47.9	37.3	82.2			
Marginal region	0	Without cord	64.9	45.2	34.4	85.4	0.678	<0.001 ** (1 mm > 0.75, 0.5, 0 mm > 0.25 mm)	0.248
		With cord	63.1	54.1	37.7	88.4			
	0.25	Without cord	41.0	4.8	38.7	43.2	0.505		
		With cord	45.6	30.5	31.3	59.9			
	0.5	Without cord	60.5	27.4	47.7	73.3	0.842		
		With cord	58.0	48.6	35.3	80.7			
	0.75	Without cord	59.9	24.2	48.5	71.2	0.885		
		With cord	61.7	49.6	38.5	84.9			
	1.0	Without cord	100.1	44.5	79.3	121.0	0.294		
		With cord	82.0	61.8	53.1	111.0			
Axial region	0	Without cord	36.1	35.3	24.9	57.2	0.541	<0.001 ** (1 mm > 0.75, 0.5, 0 mm > 0.25 mm)	0.329
		With cord	48.6	39.4	30.2	67.0			
	0.25	Without cord	29.7	3.2	28.2	31.2	0.263		
		With cord	34.7	19.3	25.6	43.7			
	0.5	Without cord	38.2	21.0	28.3	48.0	0.919		
		With cord	39.1	35.3	22.6	55.6			
	0.75	Without cord	33.2	21.6	23.1	43.3	0.502		
		With cord	39.5	35.3	22.9	56.0			
	1.0	Without cord	60.5	44.5	39.7	81.3	0.771		
		With cord	56.3	45.7	34.9	77.7			
Occlusal region	0	Without cord	36.0	35.3	24.9	57.2	0.084	0.314	0.473
		With cord	40.4	37.7	22.8	58.1			
	0.25	Without cord	22.0	3.3	20.5	23.6	0.071		
		With cord	30.6	20.4	21.1	40.2			
	0.5	Without cord	32.3	18.9	23.5	41.2	0.504		
		With cord	38.3	35.1	21.9	54.7			
	0.75	Without cord	27.6	21.2	17.7	37.5	0.414		
		With cord	35.3	35.8	18.5	52.0			
	1.0	Without cord	49.3	44.3	28.6	70.0	0.914		
		With cord	50.8	44.9	29.8	71.8			

* Statistical significance between groups with and without gingival retraction cord was assessed using the Mann–Whitney U test (*p* < 0.05). The effect of finish line depth in the without cord group ** and with cord group *** was evaluated using the Kruskal–Wallis H test (*p* < 0.05).

**Table 2:** Comparison of marginal and internal fit by subgingival finish line depth with and without gingival retraction cord.

Finish Line Depth (mm)	Marginal and Internal Fit	Cord Type	Mean	SD	95% CI		*p* *
					Lower	Upper	
0	AMD	Without cord	101.1	22.7	90.5	111.7	0.034 *
		With cord	86.6	18.9	77.7	95.4	
	MG	Without cord	51.0	11.1	45.8	56.1	0.293
		With cord	47.1	11.9	41.5	52.7	
	Chamfer gap	Without cord	56.9	10.1	52.1	61.6	0.757
		With cord	57.9	11.7	52.5	63.4	
	Axial gap	Without cord	72.0	5.9	69.3	74.7	0.853
		With cord	71.6	6.8	68.4	74.8	
	Angle gap	Without cord	54.6	14.2	48.0	61.2	0.492
		With cord	58.3	19.3	49.3	67.4	
	Occlusal gap	Without cord	50.2	12.3	44.4	55.9	0.058
		With cord	59.9	18.5	51.2	68.6	
0.25	AMD	Without cord	99.2	52.4	74.7	123.8	0.229
		With cord	83.2	26.2	71.0	95.5	
	MG	Without cord	62.0	39.1	43.7	80.3	0.46
		With cord	54.5	23.0	43.7	65.2	
	Chamfer gap	Without cord	69.3	30.4	55.0	83.5	0.289
		With cord	60.4	20.9	50.6	70.2	
	Axial gap	Without cord	83.3	34.9	66.9	99.6	0.365
		With cord	75.8	10.3	71.0	80.6	
	Angle gap	Without cord	63.1	28.4	49.8	76.4	0.757
		With cord	65.9	28.3	52.6	79.1	
	Occlusal gap	Without cord	68.8	54.9	43.1	94.5	0.933
		With cord	70.2	46.0	48.6	91.7	
0.5	AMD	Without cord	146.6	30.5	132.3	160.9	<0.001 *
		With cord	82.6	18.0	74.2	91.0	
	MG	Without cord	61.4	18.5	52.8	70.1	0.048 *
		With cord	51.3	12.2	45.6	57.0	
	Chamfer gap	Without cord	60.2	12.3	54.5	66.0	0.522
		With cord	63.1	15.6	55.8	70.4	
	Axial gap	Without cord	74.9	26.3	62.6	87.2	0.125
		With cord	65.4	6.0	62.6	68.2	
	Angle gap	Without cord	68.1	17.2	60.0	76.1	0.096
		With cord	60.7	8.4	56.8	64.7	
	Occlusal gap	Without cord	69.0	21.3	59.0	78.9	0.15
		With cord	61.4	9.4	57.0	65.8	
0.75	AMD	Without cord	150.1	19.7	140.9	159.3	0.005 *
		With cord	105.4	64.6	75.2	135.7	
	MG	Without cord	66.0	9.9	61.3	70.6	0.898
		With cord	67.5	50.9	43.6	91.3	
	Chamfer gap	Without cord	73.9	32.3	58.8	89.0	0.913
		With cord	75.2	43.2	55.0	95.4	
	Axial gap	Without cord	87.3	44.4	66.5	108.1	0.387
		With cord	77.7	20.6	68.1	87.3	
	Angle gap	Without cord	73.3	19.8	64.0	82.6	0.875
		With cord	75.1	45.7	53.7	96.5	
	Occlusal gap	Without cord	72.0	20.0	62.6	81.4	0.475
		With cord	83.8	70.3	50.9	116.7	
1.0	AMD	Without cord	189.1	42.2	169.3	208.8	<0.001 *
		With cord	95.2	22.9	84.5	106.0	
	MG	Without cord	76.8	20.2	67.3	86.2	0.082
		With cord	63.1	27.4	50.3	76.0	
	Chamfer gap	Without cord	77.6	29.1	63.9	91.2	0.177
		With cord	67.9	12.0	62.2	73.5	
	Axial gap	Without cord	84.7	12.8	78.7	90.7	0.02 *
		With cord	76.9	6.5	73.9	80.0	
	Angle gap	Without cord	91.0	19.8	81.8	100.3	<0.001 *
		With cord	64.9	9.2	60.6	69.2	
	Occlusal gap	Without cord	87.4	20.5	77.8	97.0	0.012 *
		With cord	67.1	27.5	54.3	80.0	
Without cord; *p* **		AMD; <0.001 ** (1 mm > 0.75, 0.5 mm > 0.25, 0 mm)	MG; 0.011 ** (1 mm > 0.75, 0.5, 0.25 mm > 0 mm)	Chamfer gap; 0.056	Axial gap; 0.374	Angle gap; <0.001 ** (1 mm > 0.75, 0.5 mm > 0.25, 0 mm)	Occlusal gap; 0.005 ** (1, 0.75, 0.5, 0.25 mm > 0 mm)
With cord; *p* ***		AMD; 0.195	MG; 0.156	Chamfer gap; 0.168	Axial gap; 0.050	Angle gap; 0.314	Occlusal gap; 0.360

* Statistical significance between groups with and without gingival retraction cord was assessed using the Mann–Whitney U test (*p* < 0.05). The effect of finish line depth within each group (without cord **) and (with cord ***) was evaluated using the Kruskal–Wallis H test (*p* < 0.05). AMD, absolute marginal discrepancy; MG, marginal gap.

**Table 3:** Correlation analysis of marginal region trueness with marginal and internal fit parameters.

Trueness		Marginal Fit		Internal Fit			
Marginal Region		AMD	MG	Chamfer Gap	Axial Gap	Angle Gap	Occlusal Gap
Without cord	Pearson’s correlation coefficient	0.439	-	-	-	0.268	0.232
	Correlation level *	Moderate	-	-	-	Weak	Weak
	*p* **	0.001	0.184	0.246	0.134	0.007	0.02
With cord	Pearson’s correlation coefficient	-	-	-	-	0.297	0.288
	Correlation level *	-	-	-	-	Weak	Weak
	*p* **	0.548	0.387	0.275	0.056	0.003	0.004

* Correlation coefficients were classified as strong (−0.7 to −0.9 or 0.7 to 0.9), moderate (−0.4 to −0.6 or 0.4 to 0.6), or weak (−0.1 to −0.3 or 0.1 to 0.3). ** Significance was determined using Pearson’s correlation analysis; *p* < 0.05 was considered statistically significant. AMD, absolute marginal discrepancy; MG, marginal gap.

## Data Availability

The original contributions presented in the study are included in the article, further inquiries can be directed to the corresponding author.
